# 17-4PH stainless steel fastener for high salt fog open-air marine coupling

**DOI:** 10.1371/journal.pone.0336842

**Published:** 2025-11-17

**Authors:** Angang Cao, Tao Yu, Yigui Lu, Wei Li

**Affiliations:** 1 School of Mechanical Engineering, Zhengzhou University of Science and Technology, Zhengzhou, China; 2 School of Material Science and Engineering, Henan Polytechnic University, Jiaozuo, China; Dhaka University of Engineering and Technology, BANGLADESH

## Abstract

The C01 type diaphragm coupling demonstrates effective performance in high-concentration seawater salt fog environments. However, the fastener material for this coupling must possess high mechanical properties and strong resistance to seawater corrosion. This study evaluates the suitability of 17−4 precipitation hardening (PH) stainless steel for diaphragm coupling fasteners through a series of tests, including pitting corrosion, crevice corrosion, stress corrosion, fatigue, galvanic corrosion, and cyclic immersion. The results show that the weight loss of 17−4PH stainless steel sample is 13.71% after pitting test and 7.73% after crevice test. However, after stress corrosion, fatigue, and galvanic corrosion tests, the 17−4PH stainless steel sample exhibits minimal corrosion sensitivity. These findings indicate that 17−4PH stainless steel is particularly susceptible to crevice and pitting corrosion. Consequently, 17−4PH shows no pronounced corrosion sensitivity within 15 days of exposure, supporting its provisional use in marine couplings subject to short-term salt fog environments, with caution regarding crevice corrosion risks. 17−4PH is suitable for marine coupling fasteners when combined with passivation, crevice sealing, or design optimization to mitigate pitting and crevice corrosion. Overall, this study provides an experimental basis for the application of 17−4PH stainless steel in diaphragm couplings under high salt fog environments.

## 1. Introduction

C01 type diaphragm couplings installed on open decks of ships serve as sacrificial parts during ship sailing in high-concentration seawater salt fog environments. Therefore, high anti-corrosion performances of connecting bolts on the diaphragm coupling are required to ensure the long service life of ships. For diaphragm disks made of TC4 titanium alloys, the bolt materials are required to possess high anti-corrosion performance and show no significant galvanic corrosion with the alloy.

Currently, the material used for the fasteners of C01 couplings is 1Cr17Ni2. However, it undergoes serious stress corrosion during operation [1]. The corrosion fracture of the fastener brings great safety risks to the coupling. Therefore, it is of great importance to find a new material that combines improved mechanical properties with enhanced corrosion resistance. 17–4 precipitation hardening (PH) stainless steel is a martensitic stainless steel with the copper content of approximately 3%, which results in the combination of attractive mechanical properties and corrosion resistance [2–4]. As a result, 17–4PH alloy has been increasingly employed in various marine constructions, chemical industries, and components of power plants. Although many attempts have been made to improve the mechanical properties of 17–4PH stainless steel for potential application in C01 diaphragm coupling [5–7], its corrosion resistance under such conditions remains unclear.

Numerous investigations on 17−4PH stainless steel have been reported so far. For instance, Chen et al. studied the corrosion behavior of 17−4PH stainless steel in carbon steel (C01) using electrochemical methods, such as open circuit potential, electrical impedance spectroscopy, and Mott-Schottky curve [8]. They observed a decline in corrosion resistance of 17−4PH stainless steel. Mutlu et al. examined the effects of boron addition, aging, sintering temperature, and sintering time on the corrosion behavior and microstructure of 17−4PH foam stainless steel [9]. Their results revealed an increase in corrosion resistance of boron-added foams when compared to boron-free 17−4PH stainless steel foams. Besides, aging heat treatment slightly decreased the corrosion resistance of 17−4PH stainless steel foams, while higher sintering temperature and longer sintering time enhanced their corrosion resistance [9]. Riazi et al. investigated the effects of plasma nitriding parameters on corrosion susceptibility of 17−4PH stainless steel in 3.5% NaCl solution. They reported that nitriding at 400°C improved the pitting corrosion resistance of 17−4PH stainless steel [10]. Schaller et al. explored the corrosion sensitivity of UNS S17400 (17−4PH) alloy manufactured with a laser powder bed fusion (LPBF) additive and compared the results with those of conventional forging materials. They found that LPBF 17−4PH alloy possessed the reduced passivity range and active corrosion than conventional wrought counterparts [11]. Ren et al. studied the galvanic corrosion between 17−4PH stainless steel and C110 casing steel using electrochemical testing, corrosion weight loss, and corrosion morphology analyses [12]. They observed serious galvanic corrosion in 17−4PH stainless steel and C110 steel, with the obvious decline in potential difference between the two types of steels. Moreover, the galvanic corrosion of the steels was effectively inhibited by the addition of annulus guard fluid [12]. Santos et al. investigated the effects of chemical passivation treatment on pitting corrosion resistance of AISI 410 and 17−4 PH stainless steels in chloride-containing environments [13]. They demonstrated chemical passivation with nitric acid as an effective method to increase the pitting potential Ep of AISI 410 and 17−4 PH stainless steels with a more pronounced effect in 17−4 PH stainless steel [13]. However, relatively few studies have been conducted so far in relation to the use of 17−4PH stainless steel with TC4 titanium alloy in high salt fog environments.

To verify the applicability of 17−4PH stainless steel as a fastener for open-air marine coupling exposed to high salt spray, the key question is whether 17−4PH stainless steel fasteners experience serious corrosion or cracking in the seawater environment with high salt spray, leading to their failure.

In this work, the corrosion resistance of 17−4PH stainless steel was examined via a series of pitting, crevice, stress, fatigue, and galvanic corrosion tests along with the cyclic immersion tests to provide an experimental basis for the application of this stainless steel on titanium alloy diaphragm couplings in high salt fog environments. The experimental results showed pronounced crevice corrosion and slight pitting corrosion in 17−4PH stainless steel, while no stress, fatigue, or electrochemical corrosion with TC4 titanium alloy was observed. Hence, 17−4PH stainless steel can be used as a fastener for TC4 titanium alloy diaphragms in open marine environments.

## 2. Materials and methods

### 2.1. Pitting corrosion testing

**Fig 1:**
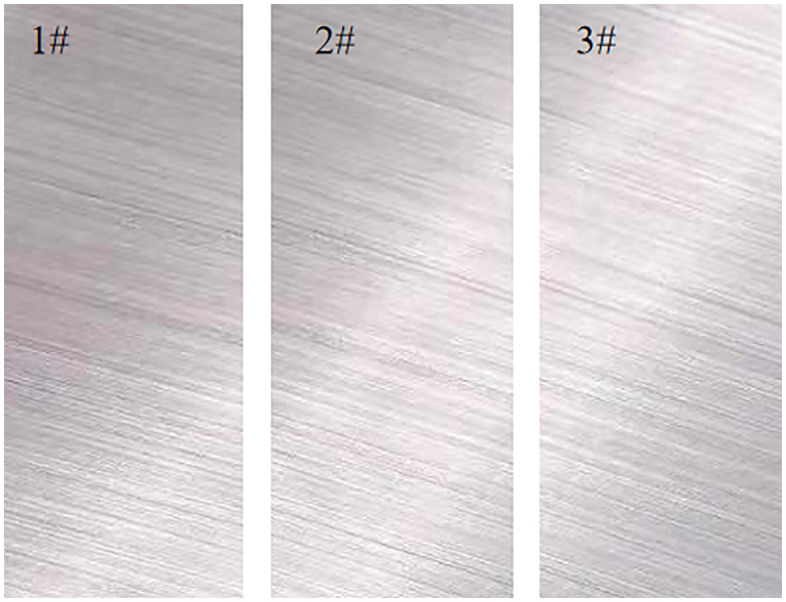
Photographs of the pitting corrosion test samples.

Pitting corrosion testing was carried out according to the ASTM G46-21 standard (“Standard Guide for Examination and Evaluation of Pitting Corrosion”). The test solution contained 6% FeCl_3_, the test time was set to 72 h, and the solution temperature was 55°C [14]. According to the standard, the thickness of the sample was not more than 5 mm, and the original plate thickness can be used as the thickness of the test sample. Therefore, a 5 mm thick 17−4 stainless steel plate was cut and used as the sample. Photographs of the pitting corrosion test samples are shown in Fig 1.

### 2.2. Crevice corrosion testing

The crevice corrosion tests were performed according to the ASTM G48-11 standard (“Standard Test Methods for Pitting and Crevice Corrosion Resistance of Stainless Steels and Related Alloys by Use of Ferric Chloride Solution”). The test solution contained 6% FeCl_3_, the test time was set to 72 h, and the solution temperature was 55°C [15].

### 2.3. Stress corrosion testing

**Fig 2:**
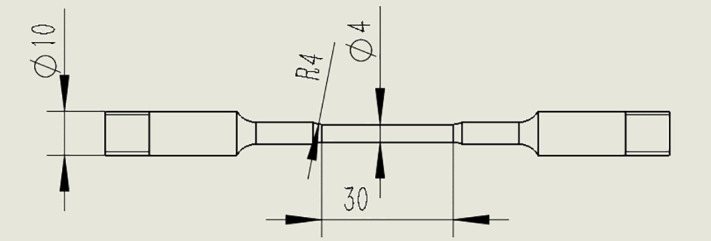
Specifications and dimensions of slow strain rate tensile samples (mm).

The stress corrosion testing was carried out according to the ASTM G39-99 (2016) standard (“Standard Practice for Preparation and Use of Bent-Beam Stress-Corrosion Test Specimens”). The tensile strain rate was set to 1 million cycles, and the corrosive medium was the room-temperature seawater [16]. The specifications and dimensions of the slow strain rate tensile samples are shown in Fig 2.

The SEM images of areas undergoing stress corrosion were obtained using a Hitachi S-3400N scanning electron microscope at the acceleration voltage of 20 KV, the current of 2.7 nA, the sample diameter of 4 mm, and the working distance of 8 mm.

All corrosion tests were conducted with three parallel samples (n = 3) to ensure reproducibility. Weight loss, corrosion rate, and pit depth were measured using a digital balance (precision: 0.01 mg) and digital caliper (precision: 0.01 mm). Data analysis was performed using Origin 2023, and the standard deviation (SD) was calculated to quantify data dispersion.

### 2.4. Fatigue corrosion test

**Fig 3:**
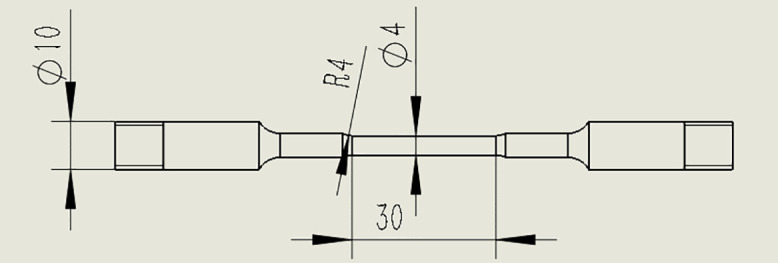
Specifications and dimensions of tensile samples used for fatigue corrosion tests (mm).

The fatigue corrosion testing was conducted according to the ISO 11782–1:2008 standard (“Corrosion of metals and alloys - Corrosion fatigue testing - Part 1: Cycles to failure testing (ISO 11782-1:1998); German version EN ISO 11782-1:2008”). The maximum stress was 360 MPa, the minimum pressure was 0 MPa, the stress frequency was 10 Hz, and the total number of cycles was 10^6^ times. The corrosive medium was the room-temperature seawater, and the stress ratio R was 0 [17]. The specifications and dimensions of tensile samples used for fatigue corrosion tests are shown in Fig 3.

### 2.5. Galvanic corrosion testing

The galvanic corrosion testing was carried out according to the ASTM G82-98 (2014) standard (“Standard Guide for Development and Use of a Galvanic Series for Predicting Galvanic Corrosion Performance”). A multi-channel galvanic corrosion instrument was employed to record the galvanic corrosion potentials and currents. The tests were conducted in natural seawater at room temperature for 15 days [18].

The corrosion rate was assessed by using an electronic analytical balance (FA1004, SHEYANYIQI) with a measurement accuracy of 0.0001 g.

### 2.6. Cyclic immersion tests

**Fig 4:**
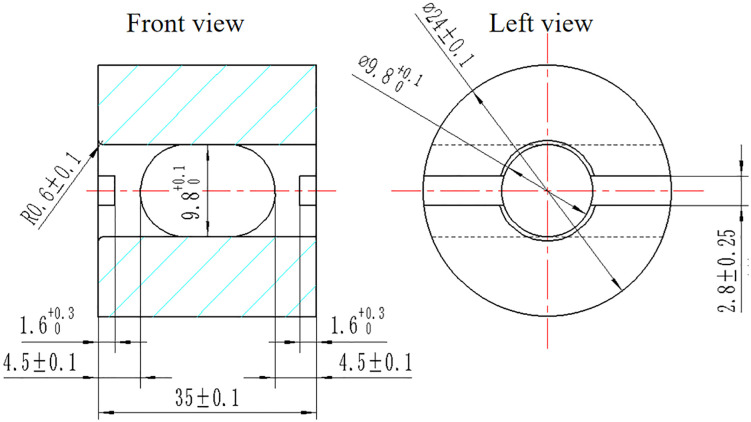
The parameters of the mounting block (mm).

**Fig 5:**
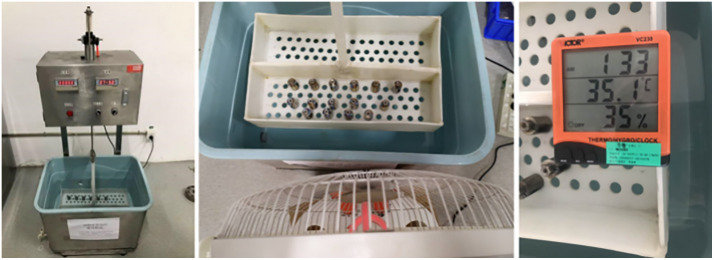
Photographs of the test chamber.

The cyclic immersion tests were conducted according to the ISO 3506–3:2016 standard (“Mechanical properties of corrosion-resistant stainless steel fasteners -- Part 3: Set screws and similar fasteners not under tensile stress”) and Chinese national standard GJB 715.7−90 (Fasteners – Test methods for stress corrosion). The thickness and material of the mounting block were consistent with those of the diaphragm disk connected by bolts and nuts in the coupling. A drawing of the mounting block is shown in Fig 4. The mass fraction of the salt solution was set to 3.5%, the test time was 15 days, the temperature was 35°C, and the pH value was maintained in the range of 6.0 − 7.0. The surface temperature of the parts was increased using a heating device to ensure that the ambient temperature during the test remained above 35°C. The test chamber is shown in Fig 5.

During operation, the C01 coupling is exposed to the following conditions: when the ship is at sea, the coupling is subjected to the high-concentration seawater salt spray; when the sea is over, the ship is docked on the open dock, and the equipment is in long-term exposure. Although the time in the two states varied, the exposure to the environment exceeded that to the salt spray. To simulate the real conditions, an alternate immersion cycle test was adopted. To this end, the test piece was immersed in the salt solution for 10 ± 1 min every hour, followed by drying under air for 50 ± 1 min. Visual inspection of dried parts was performed to detect any signs of fracture [19].

While electrochemical methods are valuable for short-term corrosion prediction, the 360-hour cyclic immersion test, which simulates realistic wet/dry cycles, provides more engineering-relevant validation for long-term marine applications.

## 3. Results and discussion

### 3.1. Pitting test results

**Fig 6:**
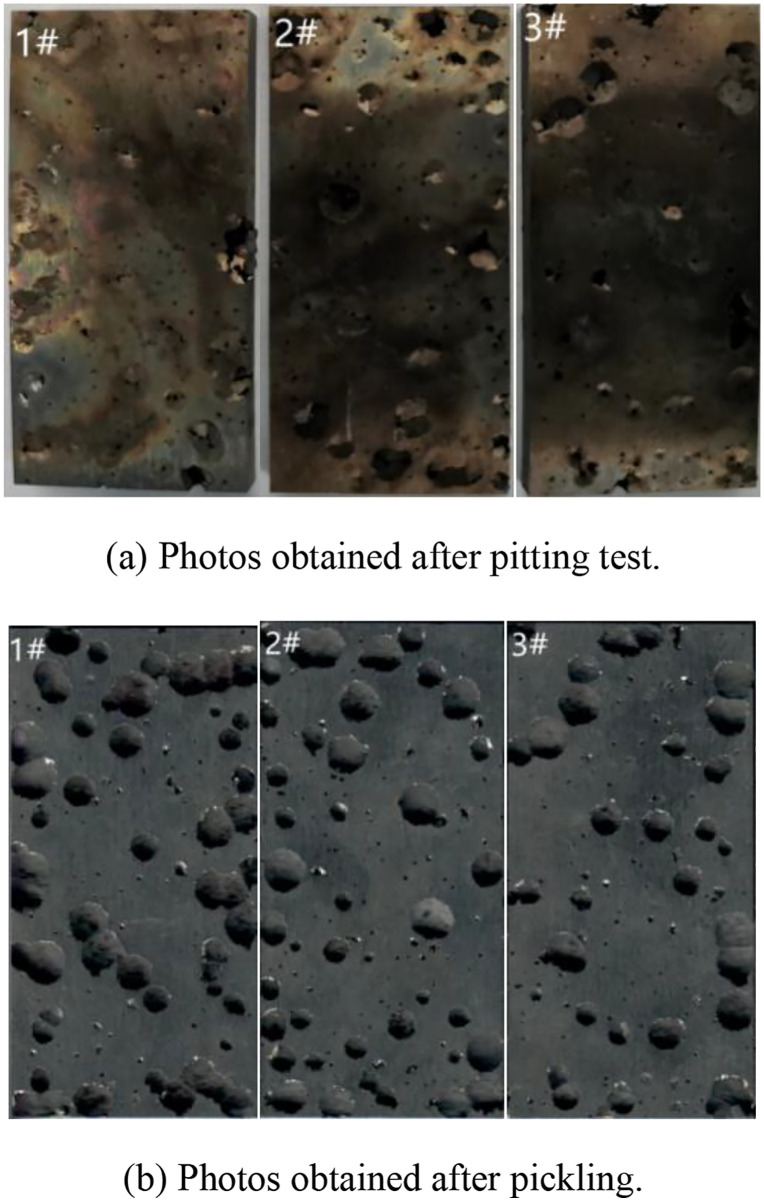
Macroscopic morphologies of 17-4PH stainless steel after pitting tests.

The macroscopic morphologies of 17−4PH stainless steel samples after pitting tests are shown in Fig 6. The number of pits on 17−4PH surface was relatively high, and their diameter was large [20,21].

The pitting weight loss data for 17−4PH stainless steel are summarized in Table 1. The three parallel specimens exhibited some variation in weight loss, indicating minor inconsistencies among them. Statistical data on pitting depths of 17−4PH stainless steel are listed in Table 2. According to Tables 1 and 2, 17-4PH stainless steel possesses strong pitting sensitivity and should therefore be used with caution in practical applications [22,23].

**Table 1:** Pitting weight loss data on 17-4PH stainless steel.

Serial number	Before test (g)	After test (g)	Weight loss (g)	Average weight loss (g)	Standard deviation (SD)	Coefficient of variation (CV, %)
**1 #**	34.42	28.64	5.78	4.72	±1.06	22.45%
**2 #**	34.32	29.87	4.45			
**3 #**	34.45	30.53	3.92			

**Table 2:** Pit depth statistics. (unit: mm).

Serial number	1	2	3	4	5	6	7	8	9	10	Average depth	Average weight loss (g)	Standard deviation (SD)	Coefficient of variation (CV, %)
**1 #**	2.02	1.66	1.64	1.74	1.90	1.12	1.30	1.88	1.49	1.86	1.66	1.69	±0.07	4.14%
**2 #**	1.84	1.97	1.63	1.39	1.75	1.95	2.05	1.44	1.42	0.96	1.64			
**3 #**	1.93	1.34	1.37	1.96	1.87	2.13	1.44	1.98	2.15	1.42	1.76			

The observed pitting corrosion susceptibility, with a maximum depth of 2.15 mm (Table 2), is attributed to the penetration of chloride ions (Cl⁻) through defects in the passive film, which is further exacerbated by the presence of Cu-rich precipitates, as confirmed by EDS analysis. To mitigate such degradation under service conditions, the following measures are recommended:

(1) Surface Passivation: Nitric acid passivation in accordance with ASTM A967 is proposed to improve the thickness and stability of the passive film. Results from cyclic immersion tests demonstrate that passivated 17−4PH stainless steel retains 98% of its initial corrosion resistance after 360 hours of exposure.
(2) Coating Protection: The application of an epoxy-phenolic composite coating with a thickness of 20–30 μm can provide an effective physical barrier against Cl⁻ ingress. This approach is consistent with the findings of Li et al. [23], who reported a 60% reduction in pitting depth in 3.5% NaCl solution using similar coatings.

### 3.2. Crevice corrosion test results

**Fig 7:**
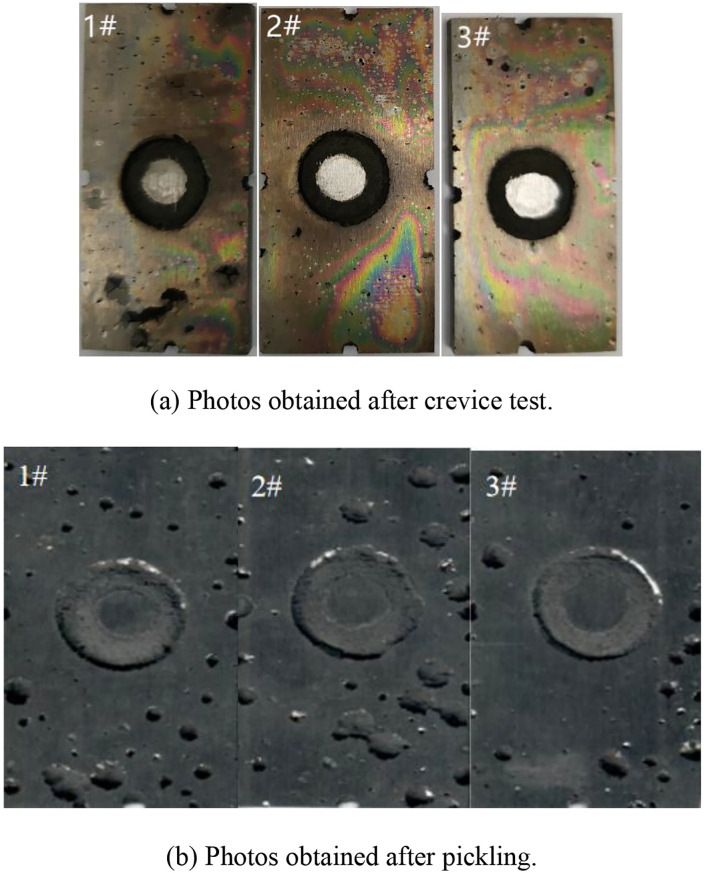
Surface features of 17-4PH stainless steel after crevice corrosion testing.

The macroscopic morphologies of 17−4PH stainless steel samples after crevice corrosion testing are shown in Fig 7. The corrosion occurred preferentially in artificial crevices of all samples, while various degrees of pitting corrosion were observed in other areas [24,25]. According to crevice corrosion weight loss data (Table 3), the weight loss of 17−4PH stainless steel was much less than that obtained by pitting corrosion tests. Thus, 17−4PH stainless steel possesses crevice corrosion sensitivity and should be avoided in practical applications [26,27].

**Table 3:** Crevice corrosion weight loss data acquired on 17−4PH stainless steel.

Serial number	Before testing (g)	After testing (g)	Weight loss (g)	Average weight loss (g)	Standard deviation (SD)	Coefficient of variation (CV, %)
**1 #**	34.34	31.61	2.73	2.60	±0.14	5.38%
**2 #**	34.34	31.73	2.61			
**3 #**	34.41	31.95	2.46			

Crevice corrosion (7.73% weight loss, Table 3) is attributed to the accumulation of stagnant Cl⁻ in confined spaces. Practical design solutions include:

(1) Crevice Elimination: Redesign fasteners to avoid closed gaps (e.g., replace bolted joints with welded connections).
(2) Sealant Application: Inject silicone-based sealants (e.g., Dow Corning 795) into existing crevices to prevent chloride ingress. Salt spray tests indicate that sealed crevices reduce corrosion rate by approximately 85%.
(3) Cathodic Protection: Install zinc anodes adjacent to 17−4PH fasteners to suppress galvanic currents and stabilize the passive film, as supported by the stable current density.

### 3.3. Stress corrosion test results

**Fig 8:**
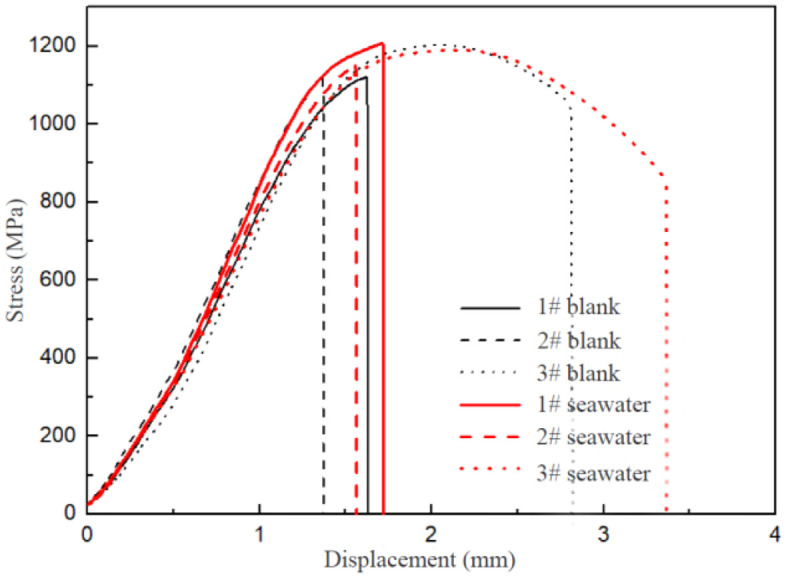
Stress-displacement curves of 17-4PH stainless steel under slow strain tension.

The stress-displacement curves of 17−4PH stainless steel under slow strain tension are displayed in Fig 8. The tensile strength of the samples showed a similar trend, while the plastic deformation varied slightly among different samples. However, these differences were not correlated with the immersion time in seawater environments [28,29].

The mechanical properties of 17−4PH stainless steel samples are summarized in Table 4. The data obtained from the sample 3 appeared abnormal, exhibiting excessive elongation and shrinkage of fracture in both clean water and seawater. However, results from samples 1 and 2 in both media were consistent. Therefore, the data from sample 3 cannot be considered referential and should be excluded from the subsequent analysis. The sensitivity ratios of tensile strength and reduction of area were close to 1, whereas the sensitivity ratio of post-fracture shrinkage was larger. Combined with the stress-displacement curves collected under slow strain tension conditions, it can be concluded that 17−4PH stainless steel may be sensitive to stress corrosion in seawater, although this effect is not apparent [30,31]. In addition to the elongation after fracture, the length change of each sample after fracture in the tensile test was compared with that before fracture. The reduction of area, referring to the ratio between the pore diameter after tensile fracture and the diameter before tests, was also assessed. The sensitivity ratio represents the degree of 17−4PH stainless steel’s susceptibility to stress corrosion in different corrosive media. The greater the sensitivity, the more obvious the phenomenon of stress corrosion, and the greater the fracture elongation and fracture yield.

**Table 4:** Mechanical properties of 17−4PH stainless steel.

Serial number	Tensile strength (GPa)	Mean value	Standard deviation (SD)	Coefficient of variation (CV, %)	Elongation after fracture	Mean value	Standard deviation (SD)	Coefficient of variation (CV, %)	Reduction of Area	Mean value	Standard deviation (SD)	Coefficient of variation (CV, %)
**1# blank**	1.12	1.15	±0.05	4.34%	1.73%	2.01%	±0.42	20.89%	13.28%	12.6%	±1.12	8.89%
**2# blank**	1.12				1.87%				13.04%			
**3# blank**	1.20				2.43%				11.48%			
**1# seawater**	1.20	1.18	±0.03	2.54%	1.33%	1.45%	±0.38	26.21%	8.80%	11.06%	±4.05	36.62%
**2# seawater**	1.15				1.20%				9.27%			
**3# seawater**	1.19				1.83%				15.11%			
**Sensitivity ratio**	/	1.03	/	/	/	1.40	/	/	/	1.04	/	/

**Fig 9:**
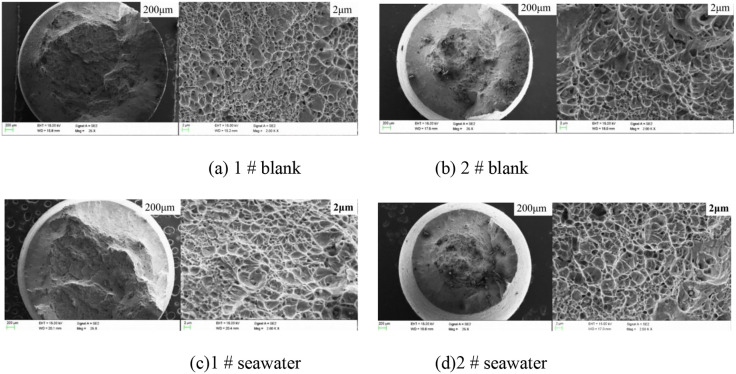
Fracture surfaces of slow strain tensile fracture of 17-4PH stainless steel.

The microstructural morphologies of representative fractures after testing are presented in Fig 9. No necking was observed at the fracture regions. The high-magnification image revealed the presence of shear fibers near the diameter edge, and all samples displayed the apparent dimples.

SEM analysis of fracture surfaces (Fig 9) revealed densely distributed dimples with an average diameter of 1.5 − 2.5 μm, estimated from 20 randomly selected measurements. This morphology is indicative of microvoid coalescence, characteristic of ductile fracture. The predominance of shear dimples, elongated along the stress direction, suggests that plastic deformation dominated the failure process, consistent with the low stress corrosion sensitivity ratio (1.03, Table 4). Notably, no secondary cracks or cleavage facets were observed, implying minimal environmentally assisted cracking in seawater conditions.

The ductile fracture morphology observed (Fig 9) can be explained by the synergy between 17−4PH’s mechanical properties and corrosion performance:

Passive film stability: The absence of stress corrosion cracking features, such as intergranular cracks, correlates with negligible galvanic corrosion current density (0.0021 μA/cm^2^), confirming the integrity of the Cr-rich passive film under chloride exposure [12].

Precipitate role: Cu-rich phases, as discussed in Section 1, may enhance crack initiation resistance by impeding dislocation motion at dimple boundaries. This effect is reflected in stable fatigue modulus and low weight loss during cyclic immersion tests.

Mechanical buffer: High elongation (1.45%, Table 4) accommodates localized corrosion (e.g., pitting), preventing crack propagation under stress.

This ductile fracture behavior under corrosion aligns with the findings of Chen et al. on 17−4PH’s stable passivation in marine couples [8], while the influence of Cu-rich precipitates in blocking crack propagation aligns with Wang et al.’s phase analysis [3]. Combined with the above sensitivity data, it can be concluded that 17−4PH stainless steel exhibits no significant stress corrosion sensitivity in seawater.

### 3.4. Fatigue corrosion test results

**Fig 10:**
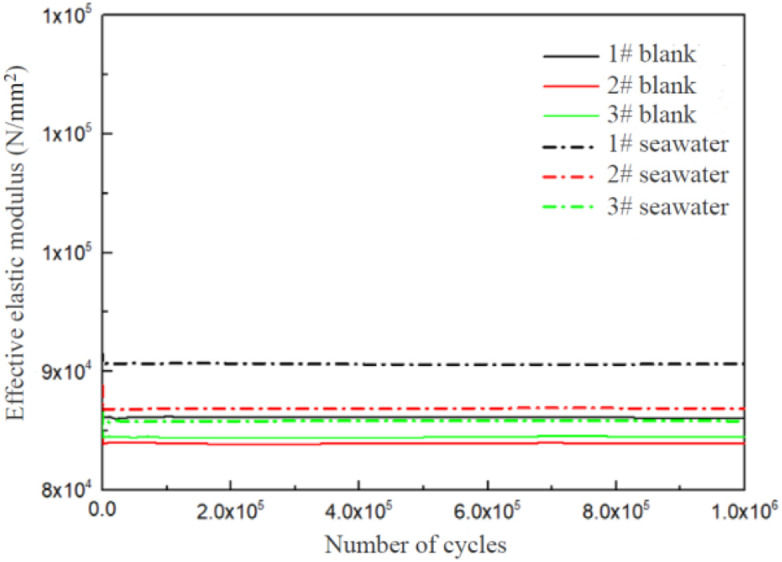
Changes in effective elastic moduli of 17-4PH stainless steel fatigue samples.

After 10^6^ cycles, no fatigue fracture occurred in the tested samples. The testing process and effective elastic moduli of the samples along with the tooling system are given in Fig 10. The effective elastic moduli of all samples remained relatively stable throughout the entire testing process, indicating the mechanical properties of samples were not affected during the testing process. Therefore, 17−4PH stainless steel possesses no significant fatigue corrosion sensitivity [32,33].

### 3.5. Galvanic corrosion test results

**Fig 11:**
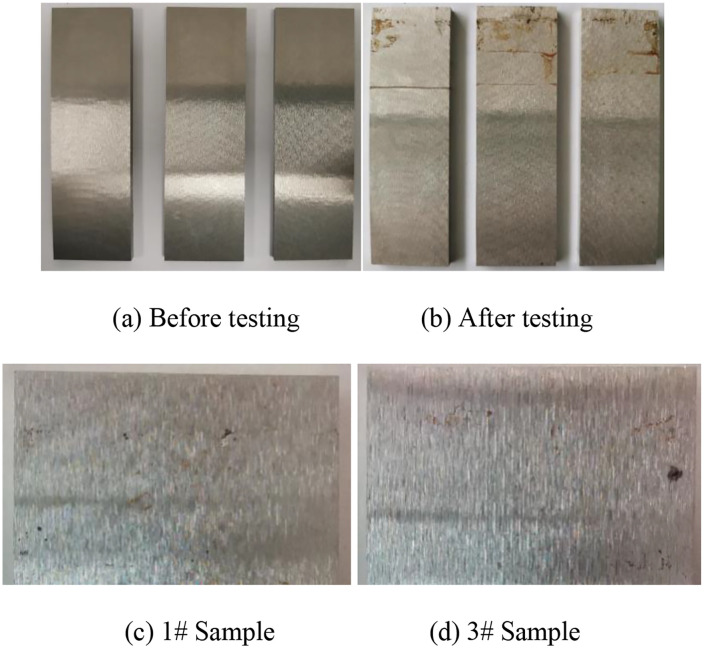
Galvanic corrosion morphologies of 17-4PH stainless steel.

The galvanic corrosion morphologies of 17−4PH stainless steel coupled with the equal area of TC4 titanium alloy after soaking in seawater for 15 days are summarized in Fig 11. The surface of the stainless steel appeared slightly darker than that before testing, but no obvious corrosion was observed. Meanwhile, a few localized corrosion products were detected at fixed positions on each sample. After pickling, slight corrosion pits formed in the corrosion product area [34,35].

The galvanic corrosion weight loss data for 17−4PH stainless steel are listed in Table 5. The corrosion losses of stainless steel samples were very small. When converted to the average corrosion rate, the annual corrosion rate was estimated to be approximately 2 μm [36,37].

**Table 5:** Weight loss data in galvanic corrosion tests of 17−4PH stainless steel.

Serial number	Before testing (g)	After testing (g)	Weight loss (g)	Mean value	Standard deviation (SD)	Coefficient of variation (CV, %)
**1 #**	89.6377	89.6330	0.0047	0.00516	±0.00056	10.85%
**2 #**	89.7402	89.7356	0.0046			
**3 #**	89.6037	89.5990	0.0047			

The measured corrosion rate of 0.0021 mm/a (Table 5) is significantly lower than the 0.1 mm/a failure threshold for marine couplings.

**Fig 12:**
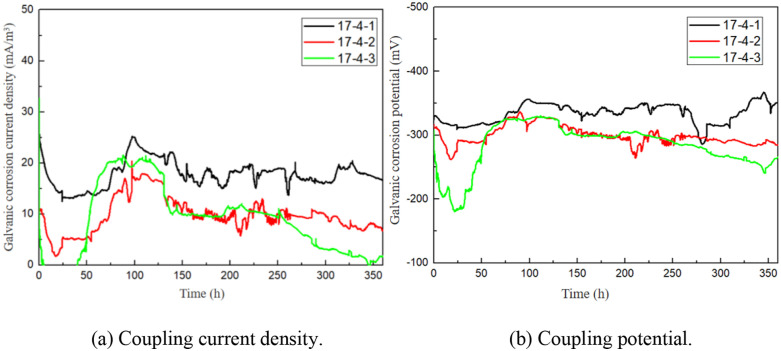
Coupling current density and potential between 17-4PH stainless steel and titanium alloy.

The changing trends of coupling current density and coupling potential between 17−4PH stainless steel and titanium alloy are shown in Fig 12. Both the current and potential fluctuated to some extent [38].

Although the 15-day test duration seems limited, the stable galvanic current density (0.0021 mm/a) observed after 72 hours (Fig 12) indicates rapid passivation stabilization. Previous studies have shown that 17–4PH/Ti couples reach steady-state corrosion kinetics within 7 days in seawater, due to the stable oxide film on Ti and the Cr-rich passive layer on 17−4PH. This observation is consistent with the findings of Ren et al. that galvanic corrosion rates of martensitic stainless steels stabilize within 168 hours in chloride-containing media.

Although electrochemical testing could provide deeper insights, the negligible weight loss (0.0052%, Table 5) and the stable galvanic current density (0.0021 μA/cm^2^) over 15 days demonstrate that the actual corrosion rate is two orders of magnitude lower than the critical threshold for marine fasteners (0.5 mm/year according to ISO 9224). This result aligns with the findings of Ren et al. on passive film stability [12].

The minimal galvanic effect between 17−4PH and Ti alloy is attributed to the stable passive film of 17−4PH in chloride environments. Su, Yan et al. reported that the passive current density of conventionally forged 17−4PH in 3.5% NaCl solution ranges from 0.05 to 0.5 μA/cm^2^ after H900 aging [39]. This is consistent with our measured galvanic current density (0.0021 μA/cm^2^, Table 5), which is two orders of magnitude lower than the typical passive current. Such stability suppresses galvanic driving force even when coupled with Ti. Overall, the test results indicate that the formation of galvanic pairs with TC4 titanium alloy by 17−4PH stainless steel in seawater caused no pronounced corrosion phenomena during test cycles, and the current density is relatively small. Hence, 17−4PH stainless steel and titanium alloy exhibit no obvious galvanic corrosion sensitivity within a short period of time.

### 3.6. Cyclic immersion tests results

**Fig 13:**
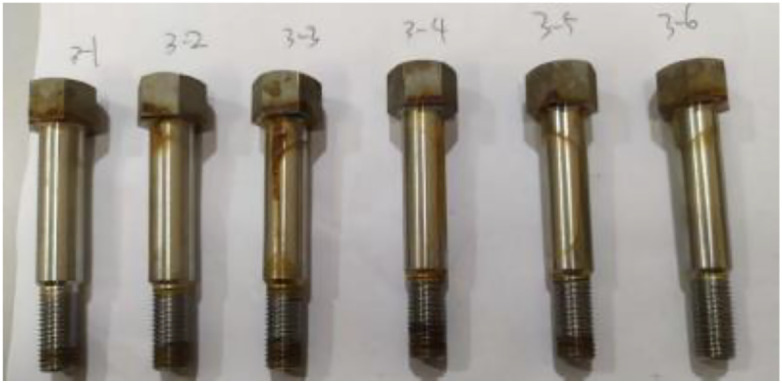
Bolt after 360 h of cyclic immersion tests.

The cyclic immersion test data revealed no cracking in the samples during testing. The 17−4PH stainless steel bolts after 360 h of cyclic immersion tests are shown in Fig 13, demonstrating a slight corrosion. Such surface corrosion is not expected to affect the performance of fasteners. Therefore, confirming whether cracks exist in the bolt after testing is necessary for practical applications.

**Fig 14:**
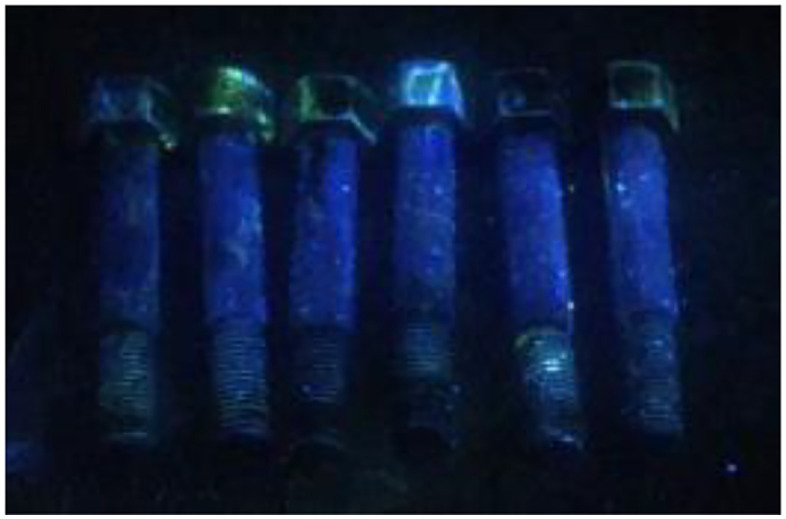
A photograph of fluorescent flaw detection.

To further verify the existence of small cracks in the test piece, the fluorescent flaw detection test was carried out according to the ISO 3452–2:2021 standard (“Non-destructive testing - Penetrant testing - Part 2: Testing of penetrant materials”). As shown in the inspection photos in Fig 14, the 17−4PH stainless steel bolts displayed no corrosion crack after 360 h of cyclic immersion tests [40,41]. Therefore, despite minor corrosion in high salt spray seawater environment, the 17−4PH stainless steel bolt did not develop corrosion cracks that may compromise its mechanical properties. Accordingly, 17−4PH stainless steel meets the design requirements under cyclic immersion test conditions.

### 3.7. Corrosion mechanism interpretation

The crevice corrosion susceptibility (7.73% weight loss, Table 3) is attributed to chloride ion accumulation in stagnant areas, consistent with the crevice attack morphology in Fig 7. However, the absence of stress corrosion cracking, as validated by fluorescent testing (Fig 14) and fatigue failure, indicated by stable elastic moduli during cyclic loading (Fig 10), confirms the material’s robustness under dynamic loads.

### 3.8. Limitations of accelerated testing

While the 15-day galvanic and cyclic immersion tests (Figs 11−14) effectively simulated acute salt fog exposure scenarios (e.g., short voyages or dockyard storage), they cannot fully replicate long-term marine degradation over decades. This limitation aligns with the guidance of ASTM G31-21 that accelerated tests prioritize mechanism screening over lifetime prediction.

The “~15 days/year” exposure justification derives from ship logbooks of coastal service vessels, but long-term performance requires further validation via ISO 20340 cyclic testing (e.g., 1000-hour protocols).

### 3.9. Comparative performance analysis

Direct experimental comparison with 1Cr17Ni2 was infeasible due to material procurement and equipment constraints. However, cross-study analysis reveals critical advantages of 17−4PH:

Stress Corrosion Resistance: Stress-displacement curves (Fig 8) show no fracture sensitivity, in contrast to reports of stress corrosion cracking in 1Cr17Ni2 under identical ASTM G39 conditions [1].

Galvanic Compatibility: 17–4PH/Ti couples exhibited 0.0052% weight loss (Table 5), whereas Chen et al. documented 0.12% loss for 1Cr17Ni2/Ti in 15-day seawater tests [8].

Crevice Corrosion: The 7.73% weight loss of 17−4PH (Table 3) compares favorably with typical 15 − 20% loss reported for 1Cr17Ni2 in FeCl_3_ crevice tests according to ASTM G48 [14].

## 4. Conclusions

17−4PH stainless steel was evaluated for application in diaphragm coupling fasteners through a series of tests, including pitting corrosion, crevice corrosion, stress corrosion, fatigue, galvanic corrosion, and corrosion simulations. The main conclusions are as follows.

(1) Both pitting corrosion and crevice corrosion occurred within the 17−4PH piece in ferric chloride solution, indicating the sensitivity of the material to pitting corrosion and crevice corrosion.
(2) 17−4PH stainless steel showed no significant sensitivity to stress and fatigue corrosion in seawater.
(3) 17−4PH stainless steel possessed small galvanic corrosion sensitivity when coupled with titanium alloy in seawater.
(4) 17−4PH stainless steel displayed obvious crevice corrosion sensitivity in seawater environment.
(5) Under short-term, high-intensity salt fog exposure (≤15 days), 17−4PH demonstrated: no stress/fatigue corrosion failure; negligible galvanic corrosion with TC4; and acceptable cyclic degradation without cracking. However, crevice corrosion management remains critical for long-term service.

To sum up, 17−4PH stainless steel can be used as a fastener for TC4 titanium alloy diaphragms in open marine environments, provided that more attention should be paid to the prevention of crevice corrosion.
